# Crowding Effects on the Structure and Dynamics of the Intrinsically Disordered Nuclear Chromatin Protein NUPR1

**DOI:** 10.3389/fmolb.2021.684622

**Published:** 2021-07-05

**Authors:** Alessio Bonucci, Martina Palomino-Schätzlein, Paula Malo de Molina, Arantxa Arbe, Roberta Pierattelli, Bruno Rizzuti, Juan L. Iovanna, José L. Neira

**Affiliations:** ^1^ CERM & Department of Chemistry “Ugo Schiff”, University of Florence, Sesto Fiorentino (Florence), Italy; ^2^ NMR Laboratory, Centro de Investigación Príncipe Felipe, Valencia, Spain; ^3^ Centro de Física de Materiales (CFM), CSIC-UPV/EHU, San Sebastián, Spain; ^4^ IKERBASQUE-Basque Foundation for Science, Bilbao, Spain; ^5^ CNR-NANOTEC, Licryl-UOS Cosenza and CEMIF.Cal, Department of Physics, University of Calabria, Rende, Italy; ^6^ Instituto de Biocomputación y Física de Sistemas Complejos (BIFI), Joint Units IQFR-CSIC-BIFI and GBsC-CSIC-BIFI, Universidad de Zaragoza, Zaragoza, Spain; ^7^ Centre de Recherche en Cancérologie de Marseille (CRCM), INSERM U1068, CNRS UMR 7258, Aix-Marseille Université and Institut Paoli-Calmettes, Parc Scientifique et Technologique de Luminy, Marseille, France; ^8^ IDIBE, Universidad Miguel Hernández, Elche (Alicante), Spain

**Keywords:** crowders, nuclear protein 1, intrinsically disordered protein, electron paramagnetic resonance, NMR, spin labelling

## Abstract

The intracellular environment is crowded with macromolecules, including sugars, proteins and nucleic acids. In the cytoplasm, crowding effects are capable of excluding up to 40% of the volume available to any macromolecule when compared to dilute conditions. NUPR1 is an intrinsically disordered protein (IDP) involved in cell-cycle regulation, stress-cell response, apoptosis processes, DNA binding and repair, chromatin remodeling and transcription. Simulations of molecular crowding predict that IDPs can adopt compact states, as well as more extended conformations under crowding conditions. In this work, we analyzed the conformation and dynamics of NUPR1 in the presence of two synthetic polymers, Ficoll-70 and Dextran-40, which mimic crowding effects in the cells, at two different concentrations (50 and 150 mg/ml). The study was carried out by using a multi-spectroscopic approach, including: site-directed spin labelling electron paramagnetic resonance spectroscopy (SDSL-EPR), nuclear magnetic resonance spectroscopy (NMR), circular dichroism (CD), small angle X-ray scattering (SAXS) and dynamic light scattering (DLS). SDSL-EPR spectra of two spin-labelled mutants indicate that there was binding with the crowders and that the local dynamics of the C and N termini of NUPR1 were partially affected by the crowders. However, the overall disordered nature of NUPR1 did not change substantially in the presence of the crowders, as shown by circular dichroism CD and NMR, and further confirmed by EPR. The changes in the dynamics of the paramagnetic probes appear to be related to preferred local conformations and thus crowding agents partially affect some specific regions, further pinpointing that NUPR1 flexibility has a key physiological role in its activity.

## Introduction

IDPs have a dynamic nature that cannot be described with a single conformation. They are mainly involved in protein-protein and protein-nucleic acid interactions which modulate several biological processes, such as transcriptional regulation, cell cycle control, replication, differentiation and RNA processing (Xie et al., 2007; Gsponer et al., 2008; Babu et al., 2011; Uversky, 2013; Wright and Dyson, 2015; Berlow et al., 2018). Usually, IDPs are engaged in multivalent and/or promiscuous interactions; in fact, hub proteins involved in the nodes of interaction networks, modulating the signals of several protein pathways, have a large proportion of disorder in their sequences (Hu et al., 2017). In spite of their dynamic nature, IDPs are far from being fully disordered: they have significant structural heterogeneity (Konrat, 2015; Kurzbach et al., 2017), which could be modulated by changes in the molecular environment, binding to other macromolecules (normally, their natural partners), or by post-translational modification (PTM)—often, but not exclusively, in the form of phosphorylation (Iakoucheva et al., 2004).

One of the main factors affecting the dynamics and the structural behaviour of IDPs is the surrounding environment. At the physiological level, the intracellular space is populated by a great number of large biological macromolecules (Theillet et al., 2014). For example, various mammalian cells revealed protein and nucleic acid concentrations in the range of 50–250 and 20–50 mg/ml, respectively, occupying up to 40% of cell volume (Theillet et al., 2014). Therefore, the restricted cellular environment can influence the flexibility and the conformation ensemble of disordered proteins (Minton, 2000; Rivas and Minton, 2016). For these reasons, different IDPs have been analysed in the presence of crowders (such as poly-ethylenglycol, Ficoll, and Dextran polymers) as these agents can simulate the crowding conditions in the internal spaces of prokaryotic and eukaryotic cells (Qu and Bolen, 2003; Roque et al., 2007; Breydo et al., 2014; Banks et al., 2018).

NUPR1 (UniProtKB O60356) is a basic, 82-residue-long (Supplementary Figure S1), monomeric IDP (Mallo et al., 1997; Chowdury et al., 2009). It does not have a stable secondary and tertiary structure, and it possesses large flexibility, which is subtly altered in the presence of its molecular partners (Neira et al., 2019). NUPR1 intervenes in chromatin remodeling and transcription, and it is an essential element in cell-cycle regulation and stress-cell response (Goruppi and Iovanna, 2010; Cano et al., 2011). It is also implicated in apoptosis through the formation of a complex with prothymosin *a* (Malicet et al., 2006), as well as intervening in DNA binding and repair (Encinar et al., 2001; Aguado-Llera et al., 2013), and in the interaction with Polycomb group proteins (Santofimia-Castaño et al., 2017). Moreover, NUPR1 also interacts with proteins involved in the nuclear transportation machinery, where phosphorylation at residue Thr68 modulates its transport into the nucleus driven by a helical conformational switch (Lan et al., 2020; Neira et al., 2020). As PTMs can alter the local structure of NUPR1 (but not its overall disordered nature), we wondered whether changes in the solution conditions (in particular, in a crowded environment) could alter its flexibility and/or its local conformation. In fact, only a limited number of studies of IDPs in the presence of crowders have been reported in the literature (Breydo et al., 2014; Bai et al., 2017; Stadmiller and Pielak, 2020) (see also (Fonin et al., 2018) and references therein) and how a crowded environment changes the physical properties of folded globular proteins and IDPs remains so far poorly understood (Stadmiller and Pielak, 2020).

In this work, we explored the crowding effects on NUPR1 by using Ficoll-70 and Dextran-40, two well-known compounds widely used to mimic cellular constituents (Fonin et al., 2018). These two crowders have been exploited in our study because they are commonly used in such types of experiments and they have larger sizes when compared to other osmolytes (see *Discussion*). We used different biophysical techniques, namely electron paramagnetic resonance spectroscopy (EPR), nuclear magnetic resonance spectroscopy (NMR), circular dichroism (CD), small angle X-ray scattering (SAXS) and dynamic light scattering (DLS), to find out the effects of these molecular crowders on the protein conformation. We used EPR spectroscopy in combination with the site directed spin labelling technique to probe the local dynamics of NUPR1 in the presence of both polymers, and then, we exploited CD, NMR spectroscopies, and SAXS and DLS scattering techniques to characterize the global conformation of the system in the presence of Ficoll-70 and Dextran-40. In fact, at the best of our knowledge, this is the first time that this protein has been studied by EPR. Our CD and NMR results show that NUPR1 in the presence of both crowders remained disordered, forming fuzzy complexes (Fuxreiter, 2018, 2020) with both of them, although local changes were observed at both protein termini, as shown by EPR. In conclusion, NUPR1 keeps its dynamic behavior also in a crowded milieu, suggesting a functional role of its intrinsic flexibility.

## Materials and Methods

### Materials

Ampicillin and isopropyl-β-D-1-tiogalactopyranoside were obtained from Apollo Scientific (Stockport, United Kingdom). Imidazole, bovine serum albumin (BSA), Trizma base, TSP ((trimethylsilyl)-2,2,3,3-tetradeuteropropionic acid), SIGMA-FAST-EDTA-free Protease inhibitor, 2,2,2-trifluorethanol (TFE), the ^15^NH_4_Cl salt and His-Select HF nickel resin were from Sigma-Aldrich (Madrid, Spain). Triton X-100, the paramagnetic probe S-(1-oxyl.2,2,5,5-tetramethyl-2,5-dihydro-1H-pyrrol-3-yl) methyl methane sulfonothioate (MTSL), ultra-pure urea, ultra-pure guanidinium hydrochloride (GdmCl), polytetrafluoroethylene (PTFE) filters with a size of 0.22 μm, and protein marker (PAGEmark Tricolor) were from VWR (Barcelona, Spain). Dextran-40, Ficoll-70 and PD-10 desalting-columns were acquired from GE Healthcare (Barcelona, Spain). Bio-Rad (Madrid, Spain) column sleeves were used for the Ni-immobility affinity column chromatography step. Amicon centrifugal devices with a cut-off molecular weight of 3 kDa were from Millipore (Barcelona, Spain). The rest of the materials were of analytical grade. Water was deionized and purified on a Millipore system.

### Design of the NUPR1 Mutants and Their Expression and Purification

Our main aim in this work was to explore how the presence of crowding agents affected the dynamics of the disordered protein NUPR1. To that end, we decided to exploit EPR spectroscopy introducing a paramagnetic tag in three different locations of the NUPR1 polypeptide chain. We thus mutated to cysteine Ala2 (at the N terminus, mutant A2C), Asn72 (at the C terminus, mutant N72C) and Leu24 (roughly in an intermediate position of the 82-residue-long polypeptide chain, mutant L24C). We avoided the introduction of the mutations in the hot-spot regions of the protein, which are located around Ala33 and Thr68, because we have previously observed that mutations at those places yielded a poor protein expression (Santofimia-Castaño et al., 2017). The positions were chosen to be far enough in the primary structure to avoid any interference with the hot spot regions of the protein. Attempts to perform further mutations were not successful, including efforts to express double mutants at those positions described above. Site directed mutagenesis on the same pet15b His-tagged vector where the wild-type NUPR1 was cloned, was carried out by NZYtech (Lisbon, Portugal).

Expression and purification of single mutants A2C, N72C, and L24C were carried out following the same protocol used for the wild-type protein (Encinar et al., 2001; Aguado-Llera et al., 2013; Santofimia-Castaño et al., 2017; Neira et al., 2019), using a BL21 (DE3) *Escherichia coli* strain. In all cases, these purifications yielded, even in the presence of SIGMA-FAST-EDTA-free protease inhibitor tablets, polypeptide chains around 2 kDa smaller than the wild-type protein, as tested by mass spectrometry and SDS-PAGE gels, likely as a consequence of protein degradation. Therefore, we changed the purification protocol: the lysis step (in the presence of 10 mM imidazole) and the washing step (in the presence of 30 mM imidazole) were carried out in the presence of 8 M urea. The last (elution) step, in the presence of 300 mM imidazole, was carried out in the absence of denaturant agent. The final polishing step, by using a gel filtration column Superdex 16/600 75 (GE Healthcare) in 50 mM buffer Tris (pH 7.5) with 150 mM NaCl, was carried out in an AKTA FPLC (GE Healthcare) by following the absorbance signal at 280 nm. Proteins were dialyzed against water, flash frozen in liquid nitrogen, and stored at −80°C until they were used. This modified purification protocol worked adequately for A2C and N72C, yielding mutants with the proper size (as concluded from SDS-PAGE analysis). The third single mutant, L24C, even in the presence of 8 M urea (or 7 M GdmCl) always led to a species with a molecular weight 2 kDa smaller than the wild-type protein. Therefore, we did not pursue any longer working with the L24C mutant.

Protein concentration was determined from the absorbance at 280 nm (ε = 2560 M^−1^ cm^−1^) of the two tyrosines (Tyr30 and Tyr36) in the mutants (Gill and von Hippel, 1989). For both mutants, protein expression was decreased tenfold when compared to that of the wild-type species. Therefore, by combining our previous experience with other mutants (Santofimia-Castaño et al., 2017) and the negative results of this work described above, we can conclude that mutation of residues at any position of the polypeptide chain of NUPR1 can be expected to yield a drastic decrease of the protein expression. The yield was even lower when working in minimal medium, which prevented the preparation of isotopically enriched samples of both mutants for NMR spectroscopy. Expression of ^15^N-labelled wild-type NUPR1 was carried out with 1  g *per* litre of media of ^15^NH_4_Cl as previously described (Santofimia-Castaño et al., 2017).

### MTSL Labelling

MTSL is a nitroxide spin radical providing the paramagnetic species that can be attached through a cysteine to a protein. For the MTSL-labelling, thawed solutions of A2C and N72C, usually in the concentration range 20–60 μM, were incubated with an excess of DTT (at a final concentration of 10 mM) for 4 h at 4°C. A PD-10 desalting column was equilibrated with 50 mM Tris (pH 7.2). The mutants were loaded in a total volume of 2.5 ml and eluted with 3.5 ml of buffer (following manufacturer instructions). Protein concentration was measured in the resulting solution, and a ten-fold excess (in molar concentration) of MTSL was added; the samples were incubated overnight at 4°C under agitation in the darkness. The following morning, a PD-10 desalting column with the same buffer described above was used to remove the MTSL excess. Protein purity was confirmed by SDS-PAGE. Samples were concentrated by using Amicon centrifugal devices (with a cut-off molecular weight of 3 kDa); the effective removal of unreacted spin label was checked by acquiring the EPR spectrum at room temperature.

### X-Band CW-EPR Spectroscopy

CW-EPR spectra of MTSL-labelled NUPR1 mutants (A2C-MTSL and N72C-MTSL) at 30 μM, in buffer (50 mM Tris, pH 7.2) and in the presence of different crowding agents were recorded on an ELEXYS E580 spectrometer (Bruker GmbH, Karlsruhe, Germany) equipped with a Super High Q resonator (SHQE) operating at X-band (9.8 GHz) at room temperature. The spectra of both mutants were recorded with a final protein concentration of 30 μM. The EPR spectrometer was set by using the following parameters: microwave power = 10 mW; magnetic field amplitude = 1 G; field sweep = 150 G; receiver gain = 60 dB; and modulation frequency = 100 kHz. To derive the spectral parameters (that is, the correlation times and the corresponding component population), all the EPR spectra were simulated with the SimLabel program (Etienne et al., 2017) (GUI of Easyspin (Stoll and Schweiger, 2006)), obtaining the indicated spectral parameters. The parameters obtained correspond to different dynamic states of the MTSL probe.

### NMR

The NMR experiments were performed at 25°C on a Bruker Avance-II 600 MHz spectrometer (14.1 T) (Bruker GmbH, Karlsruhe, Germany), equipped with a triple resonance cryogenically-cooled probehead and z-pulse field gradients. The temperature of the probe was calibrated with methanol (Cavanagh et al., 1996). The concentration of ^15^N-labeled NUPR1 was 110 μM. The buffer used in the experiments was Tris (pH 7.2, 50 mM). The peaks in the 2D^15^N, ^1^H-TROSY-HSQC-NMR spectrum (Bodenhausen and Ruben, 1980; Pervushin et al., 1997) of wild-type NUPR1 were identified by using the assignments previously determined (Aguado-Llera et al., 2013). The crowding agent concentrations were either 50 or 150 mg/mL. A control experiment was also carried out in the presence of 50 mg/ml of BSA at the same pH and temperature as the experiments in the presence of the other crowders.

The spectra were acquired with 2 K complex points in the ^1^H dimension and 128 complex points in the ^15^N dimension, with 40 scans. The spectral widths were 5,411 (^1^H) and 1,277 (^15^N) Hz. Spectra in the absence and presence of the crowders were acquired with the carrier frequency at the water signal. The resulting matrix of each experiment was zero-filled to double the number of original points in all dimensions and shifted squared sine-bell apodization functions were applied, before Fourier transformation. NMR data were processed and analyzed using TopSpin 3.2 (Bruker GmbH, Karlsruhe, Germany). Spectra were calibrated with external TSP for ^1^H and for the indirect dimensions as described (Cavanagh et al., 1996).

### CD

Far-UV CD spectra were collected on a Jasco J810 spectropolarimeter (Jasco, Tokyo, Japan) with a thermostated cell holder, and interfaced with a Peltier unit at 25°C. The instrument was periodically calibrated with (+)−10-camphorsulphonic acid. Cells with a path length cell of 0.1 cm were used (Hellma, Kruibeke, Belgium). All spectra were corrected by subtracting the corresponding baseline. The concentration of wild-type NUPR1 was 20 μM; the buffer used in the experiments was Tris (pH 7.2, 50 mM). Isothermal wavelength spectra at different concentrations of either Ficoll-70 or Dextran-40 (50, 100 and 150 mg/ml) were acquired at a scan speed of 50 nm min^−1^ with a response time of 2 s, a band-width of 1 nm, and averaged over six scans. Experiments were repeated twice at different days. Variations from day to day of the voltage of the photomultiplier, with newly prepared samples, were less than 4%. The samples were prepared the day before and left overnight at 5°C to allow for equilibration. Before starting the experiments, samples were left for 1 h at 25°C.

Control experiments were also carried out with the isolated MTSL-labelled mutants (at 30 μM of protein concentration) to be sure that labelling did not alter the conformational properties of the protein. Experiments were carried out at the same pH and temperature, as well as with the same set-up, as the experiments in the presence of crowders described above.

### Small Angle X-Ray Scattering (SAXS) and DLS

We acquired SAXS experiments to confirm that wild-type NUPR1 at these conditions (50 mM Tris buffer, pH 7.2) showed the same conformational features as those found previously under different conditions (50 mM acetic buffer, 50 mM, pH 4.5) (Neira et al., 2019). SAXS experiments were performed on a Rigaku 3-pinhole PSAXS-L equipment operating at 45  kV and 0.88 mA, and with samples at 25°C in Tris buffer (50 mM, pH 7.2) with a NUPR1 concentration of 4.3 mg/ml (∼460 μM). The MicroMax-002 + X-ray Generator Systems is composed by a microfocus sealed tube source module and an integrated X-ray generator unit that produces Cu-Kα transition photons of wavelength *λ* = 1.54 Å. The flight path and the sample chamber were under vacuum. The scattered X-rays were detected on a two-dimensional multi-wire X-ray detector (Gabriel design, 2D-2000×). The azimuthally averaged scattered intensities were obtained as a function of scattering vector Q (with Q = (4πsin (θ/2))/λ, where θ is the scattering angle). Reciprocal space calibration was done using silver behenate as standard. The solutions were filling boron-rich capillaries with an outside diameter of 2  mm and a wall thickness of 0.01  mm. The contribution from the corresponding buffer (measured on the same capillary) was subtracted by applying the proper factors obtained from transmission measurements. The sample-detector distance was 2  m, allowing the coverage of a Q-range from 0.008 to 0.2 Å^−1^. From the intensity scattered at low-Q values–in the so-called Guinier regime–we could determine the average gyration radius, *R*_g_, of the protein, by using the Guinier law as we have previously described (Díaz-García et al., 2020). Furthermore, we could also estimate the compaction of the polypeptide chain through the scaling factor, υ. The scaling factor determines the Q-dependence of the scattered intensity at intermediate Q-values and can be obtained through the fit of a generalized Gaussian coil function to the experimental results. This function is expressed as (Hammouda, 2012): $I\left(Q\right)\approx \frac{1}{\nu U^{1/2\nu }}\gamma \left(\frac{1}{2\nu },U\right)-\frac{1}{\nu U^{1/\nu }}\gamma \left(\frac{1}{\nu },U\right)$, where $U=\left(2\nu +1\right)\left(2\nu +2\right)Q^{2}R_{g}^{2}/6$ and $\gamma \left(a,x\right)=\int_{0}^{x}t^{a-1}\exp\left(-t\right)dt$. The values of υ are 1/3 for a polymeric chain collapsed into a globule; 0.5 for an unordered polymer (which is the conformation of a linear polymer chain in θ-conditions); and 0.6 for a swollen chain in a good solvent (the so-called self-avoiding-walk conformation).

The DLS experiments were carried out at 22 °C with a NUPR1 concentration of 4.3 mg/ml either in the absence or in the presence of 150 mg/ml of Ficoll-70 in Tris buffer (50 mM, pH 7.2). Control experiments were also carried out in the absence of NUPR1, with a Ficoll-70 concentration up to 150 mg/ml. DLS measurements were performed in a 3D-LS spectrometer (LS Instruments AG, Fribourg, Switzerland) equipped with a high-performance 100 mW DPSS laser with a wavelength of 660 nm. Cylindrical sample cells were placed in an index matching vat filled with decalin. Intensity autocorrelation functions were recorded at angles θ between 30° and 140°. The scattering vector was determined as Q = (4π*n*sin (θ/2))/λ,where *n* is the refractive index of the solution. The measured intensity autocorrelation function, g^2^(t), is related to the electric field autocorrelation function, |g^1^(t)|, by the Siegert relationship: g^2^(t) = 1 + B |g^1^(t)|^2^, where B is an instrumental constant close to one in our experimental set-up.

Had we a simple diffusion of the macromolecules in the measurement, the correlation function could account for a single exponential function: g^1^(t) = *A* exp (−t/τ), where τ is the relaxation time, and *Γ* = 1/τ would exhibit a Q^2^ dependence related to the collective translational diffusion of the polymer, *D*, as: *Γ* = *D*Q^2^. At infinite dilution, the polymers and the polypeptide chain can be regarded as non-interacting and *D* is related to the hydrodynamic radius, *R*_h_, through the Stokes-Einstein equation. However, in the presence of two relaxation modes, a common way of analyzing the data is fitting g^1^(t) to a multi-exponential function composed by the sum of two exponential decays for the fast relaxation and slow relaxations, respectively, which is given by:

$${\text{g}}^{1}\left(\text{t}\right)=A_{f}\cdot \exp\left(-t/\tau_{f}\right)+A_{sl}\cdot \exp\left(-t/\tau_{sl}\right)$$ (1)

where the amplitudes, *A*_i_, and the relaxation times, τ_i_, characterize the two relaxation processes. That equation described well the experimental data of the collective dynamics for the isolated NUPR1 and the isolated Ficoll-70. However, for the mixture of both species, fitting to Eq. 1 was poor, and the following expression was used:

$${\text{g}}^{1}\left(\text{t}\right)=A_{f}\cdot \exp\left(-t/\tau_{f}\right)+A_{sl}\cdot \exp{\left(-t/\tau_{sl}\right)}^{\text{β}}$$ (2)

where β characterizes the second relaxation (slow) process.

### Viscosity Measurements

The viscosities at different crowder concentrations of the solution (in the range from 20 to 150 mg/ml) were measured by using an electromagnetically spinning viscometer EMS-1,000 viscometer (Kyoto Electronics, Kyoto, Japan) operating at 22°C, following the manufacturer instructions.

## Results

### Far-UV CD

**FIGURE 1:**
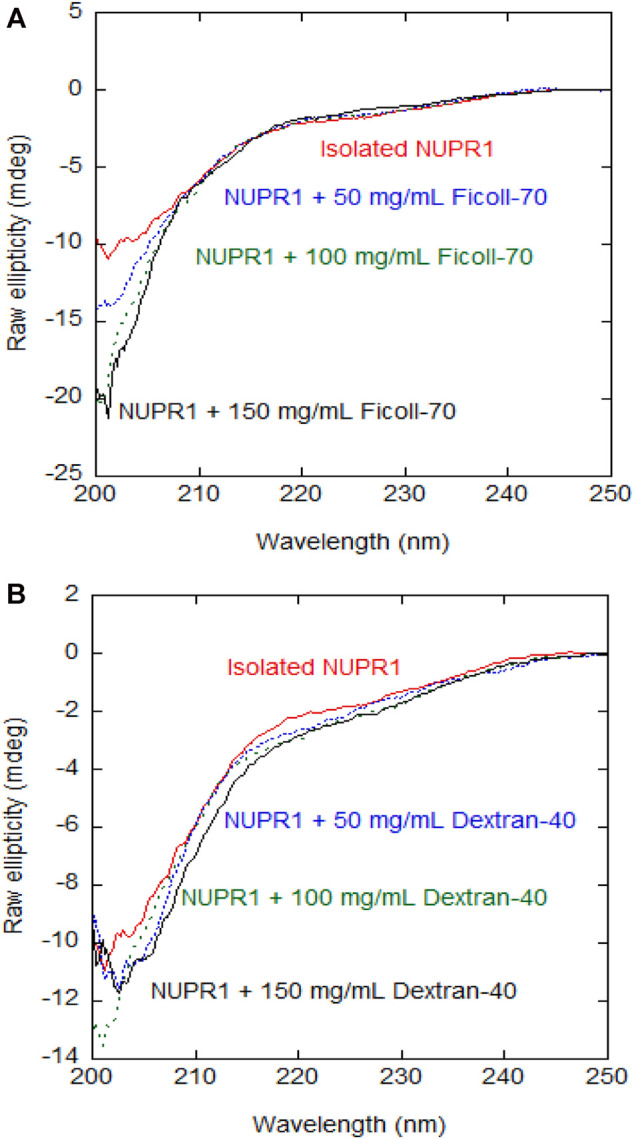
**Far-UV CD spectra of wild-type NUPR1 in the presence of crowders**: Far-UV CD spectra of wild-type NUPR1 at different concentrations of Ficoll-70 **(A)** and Dextran-40 **(B)**. Experiments were carried out in buffer Tris (50 mM, pH 7.2) at 25°C.

We preliminarily used CD at different concentrations of crowding agents to assess whether their presence increased the percentage of secondary structure in NUPR1. The far-UV CD spectra indicate that, at increasing concentrations of any of the crowders, there were no major changes in the spectra and then, in the percentage of secondary structure as it could be estimated from CD (Figure 1). The spectra of the isolated synthetic polymers are shown in Supplementary Figure S2, and since both are chiral, their far-UV CD spectra in isolation had observable signals. These findings indicate that crowders, in the concentration range explored, did not appear to alter the disordered nature of NUPR1.

Furthermore, experiments with the isolated MTSL-labelled mutants showed that the disordered features of NUPR1, as monitored by far- UV CD, were not altered with labelling (Supplementary Figure S3).

### NMR

The 2D^15^N, ^1^H- TROSY-HSQC-NMR spectrum of NUPR1 in isolation at pH 7.2 showed only a small subset of signals due to the amide signals broadening induced by the highly efficient hydrogen-exchange with solvent protons (Supplementary Figure S4A). These residues belong to the most hydrophobic region of the protein (Neira et al., 2017; Santofimia-Castaño et al., 2017). In the presence of 50 mg/ml of Ficoll-70, all the signals due to these residues (except that or Arg82) were observed, but with an overall smaller intensity than those in the spectrum of isolated NUPR1 (Supplementary Figure S4B) probably due to the higher viscosity of the sample in the presence of the crowder agent (Supplementary Figure S5). At 150 mg/ml of Ficoll-70, no residue cross-peaks were observed.

The addition of 50 mg/ml Dextran-40 produced a similar decrease in signal intensity and the disappearance of the signals of residues Thr3, Thr8, Ala10, Ser58, and Arg82 (Supplementary Figure S4C). Conversely to what happened with Ficoll-70, at 150 mg/ml of Dextran-40, we were still able to detect the signals of Gly16, Glu18, Ser22, Ser27, Leu29, Tyr30, Ser31, Leu32, and Gly38. At all the concentrations of synthetic polymers explored, the viscosity of the Ficoll-70 samples was larger than that of Dextran-40, due to the larger molecular weight of the polymer (Supplementary Figure S5) and this is likely the reason why we were still able to observe cross-peaks at 150 mg/ml of Dextran-40. The distinct behavior of the different cross-peaks of NUPR1 indicates that there must be weak, non-specific binding interactions with both crowders (Pastore and Temussi, 2017). In any case, none of the crowders changed the disordered nature of NUPR1, thus confirming the results obtained by far-UV CD (Figure 1).

As it is a matter of debate whether crowders are good mimickers of physiological-like conditions, we obtained a control 2D 15N, 1H-TROSY-HSQC-NMR spectrum of NUPR1 in the presence of 50 mg/ml of BSA. We believe we can mimic a more physiological-like environment, in the presence of a higher concentration of the total amount of crowding protein surrounding NUPR1. Our results (Supplementary Figure S6) indicate that the behaviour observed in the broadening of signals was similar to that observed with both the other crowders. BSA has been also shown to be a good mimicker of physiological-like conditions for other IDPs, such as *α*-synuclein (Theillet et al., 2016), which also remains disordered in crowded conditions.

### EPR

To get insights about how the flexibility of the protein was altered in the presence of the synthetic polymers, we applied EPR spectroscopy.

EPR spectroscopy coupled with SDSL technique is a powerful method to analyze the conformation and the dynamics of proteins (Klare, 2013; Altenbach et al., 2015) and others macromolecules, such as DNA, RNA and lipids (Subczynski et al., 2010; Shelke and Sigurdsson, 2012); in some cases, it detects minor or transient populations, otherwise difficult to reveal with other biophysical methods (such as NMR, X-ray crystallography or cryo-electron microscopy). Specifically, nitroxide spin labels (at X-band, with a microwave frequency of 9.8 GHz) was demonstrated to be an excellent tag to probe the environment surrounding the labelled position (Bordignon, 2017). By deriving the EPR spectral parameters from simulation, and in particular the correlation time values (τ_c_) and the component percentages, the dynamics at the local level of a macromolecule can be analyzed. A high degree of protein flexibility yields sharp EPR signals (τ_c_ < 1 ns), whereas broader EPR spectra (τ_c_ > 1 ns) are usually recorded when more compact conformations are present. For these reasons, SDSL-EPR spectroscopy with nitroxide spin label has emerged as a valid approach for the characterization of various IDPs and to check possible local disorder-to-order transitions (Kavalenka et al., 2010; Longhi et al., 2011; Palombo et al., 2017; Bonucci et al., 2020; García-Rubio, 2020).

**FIGURE 2:**
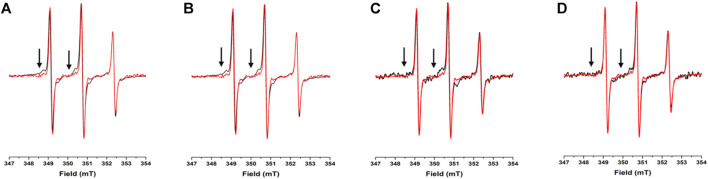
**EPR spectra under different conditions for A2C-MTSL**: X-band CW-EPR spectra at room temperature of the A2C-MTSL mutant in buffer (red) and in presence of crowders (black): Ficoll-70 at 50 mg/ml **(A)** and 150 mg/ml **(B)**, Dextran-40 at 50 mg/ml **(C)** and 150 mg/ml **(D)**.

**FIGURE 3:**
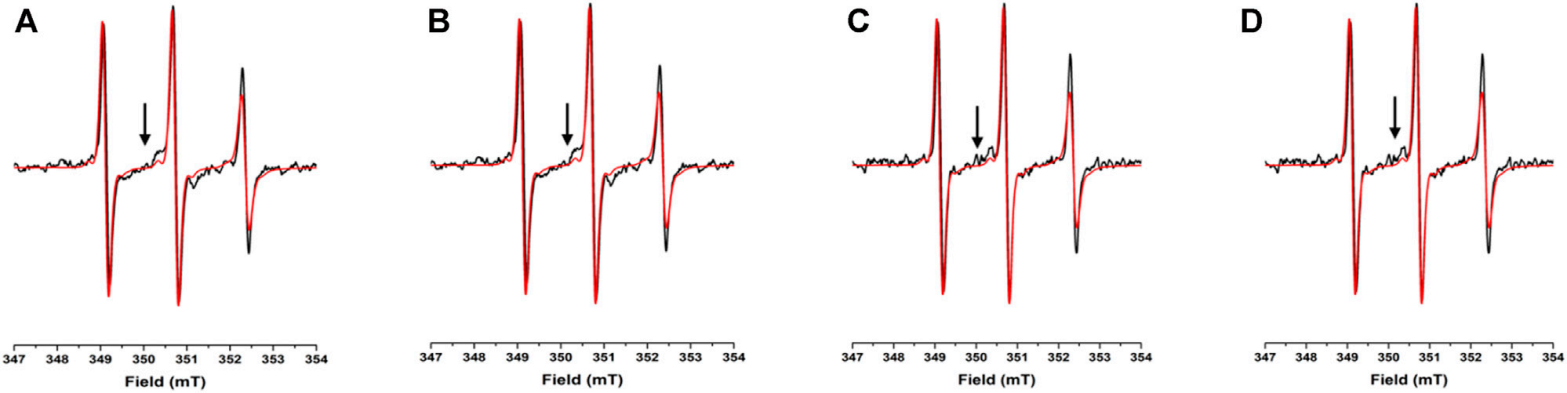
**EPR spectra under different conditions for N72C-MTSL**: X-band CW-EPR spectra at room temperature of NUPR1 N72C-MTSL in buffer (red) and in presence of crowders (black): Ficoll-70 at 50 mg/ml **(A)** and 150 mg/ml **(B)**, Dextran-40 at 50 mg/ml **(C)** and 150 mg/ml **(D)**.

To assess the potential propensity of NUPR1 to change local conformation in the presence of crowders, the EPR spectra of A2C-MTSL and N72C-MTSL samples were obtained in aqueous solution (Figures 2, 3, red line) and in the presence of the crowding agents, Ficoll-70 and Dextran-40 (Figures 2, 3, black line). The EPR spectrum of A2C-MTSL in aqueous solution showed a typical spectral line shape of a disordered protein (Longhi et al., 2011; Bonucci et al., 2020; García-Rubio, 2020). Simulation of the spectrum (Supplementary Figure S7) revealed that it is composed of a single sharp component with a fast correlation time value of 0.12 ns (Table 1; Figure 2A). These results clearly indicate that the region of the protein around Ala2 possessed a very flexible conformation *in vitro* and the N-terminal region of NUPR1 had no propensity to assume a globular conformation. Similar EPR experiments were carried out for N72C-MTSL (Figure 3 red line, Table 2). In aqueous solution the EPR spectrum of N72C-MTSL had a lineshape and a spectral composition (Supplementary Figure S8) analogue to that obtained for A2C-MTSL. All data obtained confirmed that NUPR1 was disordered in solution with the expected behaviour of an IDP at both termini.

**TABLE 1:** Correlation times (τ_c_) and component percentage (%) of the A2C-MTSL EPR spectra recorded under different solution conditions at 25°C^a^.

Conditions	Sharp component		Broad component	
—	τ_c_ (ns)	Population (%)	τ_c_ (ns)	Population (%)
Aqueous solution	0.12	100	—	—
Ficoll-70 (50 mg/ml)	0.14	47	1.69	53
Ficoll-70 (150 mg/ml)	0.15	38	2.08	62
Dextran-40 (50 mg/ml)	0.13	40	3.28	60
Dextran-40 (150 mg/ml)	0.15	40	3.30	60
Sucrose (30% v/v)	0.20	100	—	—

a Errors in the τ_c_ and the percentages of population are assumed to be 10%.

**TABLE 2:** Correlation times (τ_c_) and component percentage (%) of the N72C-MTSL EPR spectra recorded under different solution conditions at 25°C^a^.

Conditions	Sharp component		Broad component	
—	τ_c_ (ns)	Population (%)	τ_c_ (ns)	Population (%)
Aqueous solution	0.12	100	—	—
Ficoll-70 (50 mg/ml)	0.10	39	2.37	61
Ficoll-70 (150 mg/ml)	0.10	39	2.37	61
Dextran-40 (50 mg/ml)	0.10	56	1.96	44
Dextran-40 (150 mg/ml)	0.10	42	1.96	58
Sucrose (30% v/v)	0.21	100	—	—

a Errors in the τ_c_ and the percentages of population are assumed to be 10%.

For A2C-MTSL, the EPR spectra in presence of either Ficoll-70 or Dextran-40 (Figure 2 black lines) were partially broadened compared to that recorded in aqueous solution. The spectral simulations carried out for A2C-MTSL with Ficoll-70 at 50 and 150 mg/ml revealed the presence of a broad component with a τ_c_ value of 1.7 and 2.1 ns respectively (Table 1, Supplementary Figure S7). Furthermore, a sharp component analogous to that found for A2C-MTSL in solution was present. Similar results were obtained for Dextran-40 (Table 1), with a higher τ_c_ (∼3.0 ns) for the broad component when compared to the one obtained in the case of Ficoll-70. These data suggest that the N-terminal region of NUPR1 possessed both highly-dynamics and partially-rigid conformations in crowded environments.

The EPR spectra of N72C-MTSL in the presence of Ficoll-70 and Dextran-40 (Figure 3 black line) have a similar behavior to those of A2C-MTSL (Supplementary Figure S8). The spectra presented two components, a sharp one similar to that obtained in aqueous solution (Table 2) and a broad component corresponding to τ_c_ values of 2.0 and 2.4 ns for Ficoll-70 and Dextran-40, respectively (Table 2). These results clearly indicate that both crowders induced in N72C-MTSL a variation in protein dynamics, shifting the equilibrium towards a less mobile (and probably more compact) conformation than that observed in aqueous solution at the C terminus of the protein.

The use of crowders yielded an increase in solution viscosity (Supplementary Figure S5) which can strongly affect the tumbling motion of the nitroxide label attached to the protein, as it does for the whole protein (see *Far-UV CD*). Since the EPR spectral line shapes depend also on the dynamics of spin labelled side chains (Bordignon, 2017; García-Rubio, 2020; Klare, 2013), we decided to acquire EPR spectra of A2C-MTSL and N72C-MTSL in the presence of sucrose (at 30% v/v) to test the effect of viscosity (Supplementary Figure S7 and Supplementary Figure S8). Sucrose is generally considered as an osmolyte with a behavior different from typical crowders (such as Ficoll-70 or Dextran-40) (Fleissner et al., 2009). The simulations of the EPR spectra of A2C-MTSL and N72C-MTSL with sucrose showed the presence of single sharp signals with τ_c_ of 0.2 ns (Tables 1 and 2, Supplementary Figure S7 and Supplementary Figure S8), similar to that observed in aqueous solution. Therefore, we can conclude that the changes observed for spin labelled NUPR1 mutants in the presence of Ficoll-70 and Dextran-40 were induced by weak interactions between the protein and the crowders, and they are not related to an increase of viscosity.

To assess the propensity of NUPR1 to assume less flexible, local conformations, we recorded the X-band CW-EPR spectra for both variants in the presence of 25% v/v of TFE (Supplementary Figure S9). This agent is commonly used to enhance the population of *α*-helix structure in polypeptides, when they have an intrinsic propensity to acquire that type of secondary structure. Previous far-UV CD measurements (Encinar et al., 2001) on NUPR1 show an increase of the ellipticity at 222 nm (in absolute value) when the TFE concentration is raised. This seems to indicate a rise in the helical structure, although such ellipticity increase does not result in a concomitant spreading of the signals of NUPR1 in the 1D ^1^H-NMR spectra (Encinar et al., 2001). The EPR spectra in the presence of 25% TFE for A2C-MTSL (Supplementary Figure S9) did not show any variation of the spectral line shape, compared to the results obtained in aqueous solution (Figure 2 black line). In fact, the corresponding simulation indicates that the EPR signal was composed by a single component with the same τ_c_ value obtained for A2C-MTSL in aqueous solution. Conversely, N72C-MTSL mutant in the presence of 25% TFE revealed a different behavior. A broad component (∼51% of total EPR signal with τ_c_ = 1.6 ns) was detected (Supplementary Figure S9), indicating the presence of a local conformation with restricted dynamics. Summarizing these results and comparing them with those obtained previously (Encinar et al., 2001) further pinpoint that the two terminal regions of NUPR1 possess the propensity to undergo to a conformation with a reduced flexibility, without assuming a defined structure.

Finally, to assess that in the presence of BSA (and therefore in a more physiological-like environment), the behavior of NUPR1 was similar to that observed in the presence of the crowding agents for both mutants, we acquired EPR spectra at 150 mg/ml of the crowding protein (Supplementary Figure S10). For A2C-MTSL, we observed two components composing the total signal: a sharp one (31%) defined by a similar τ_c_ value of 0.16 ns; and a broader one (69%) with a τ_c_ value of 2.78 ns Similar results were obtained for N72C-MTSL suggesting that the behavior of both spin-labelled mutants is comparable to that observed in the presence of the synthetic polymers, also supported by the NMR results described above (*NMR*). In addition, we speculate that these crowders can be considered good mimickers of a physiological-like environment for NUPR1.

### SAXS and DLS

**FIGURE 4:**
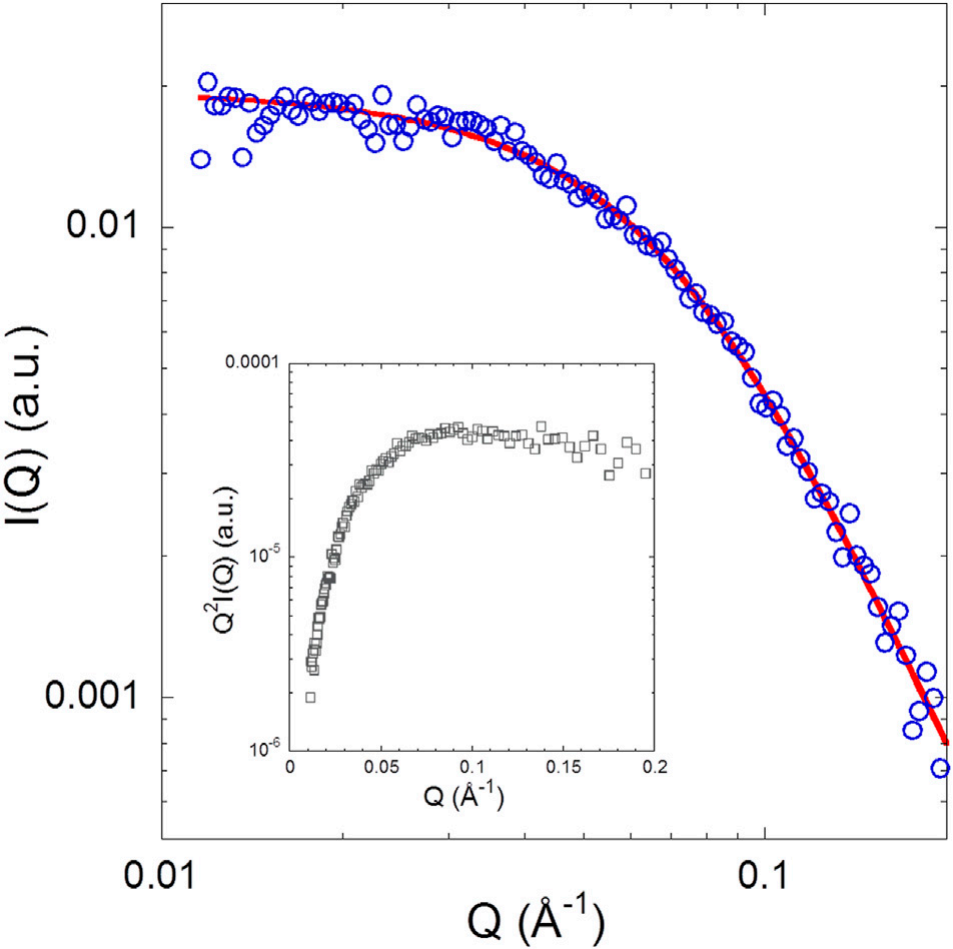
**SAXS pattern of NUPR1**: The solid line represents a fit with a generalized Gaussian coil with *R*_*g*_ = 2.5 nm and *v* = 0.36 ± 0.01. Inset: Kratky plot of the experimental data.

Figure 4 shows the SAXS pattern of isolated NUPR1 at 4.4 mg/ml (∼460 μM) concentration solution, where the form factor of a polymer coil is perfectly distinguishable. There was no evidence of aggregation (i.e., an intensity upturn at low Q values) in the length scale explored (0.01 < Q < 0.2 Å^−1^). The results are similar to what has been observed in previous measurements at pH 4.5 for isolated NUPR1 (Neira et al., 2019), as can be appreciated from the comparison of the respective Kratky plots. In the inset of Figure 4 showing such a representation, instead of the pronounced maximum corresponding to a compact globular object, a very broad curvature is observed with an almost plateau-like behavior at high Q values. A Q-independent high-Q plateau would correspond to a scaling factor of υ = 0.5. Such a random coil conformation is typical of flexible, unfolded proteins. The slight tendency of the data in the Kratky representation to decrease at high Q values indicates, however, a somewhat more compact conformation than that corresponding to υ = 0.5. The fit to a generalized Gaussian coil form factor of the scattered intensity yielded *R*_g_ = 2.5 nm (with an error of 5%), and a value of the scaling factor smaller than 0.5, namely υ = 0.36 ± 0.01. This indicates a conformation more compact than a polymer coil in θ-solvent (*υ* = 0.5), but yet not globular (*υ* = 1/3).

**FIGURE 5:**
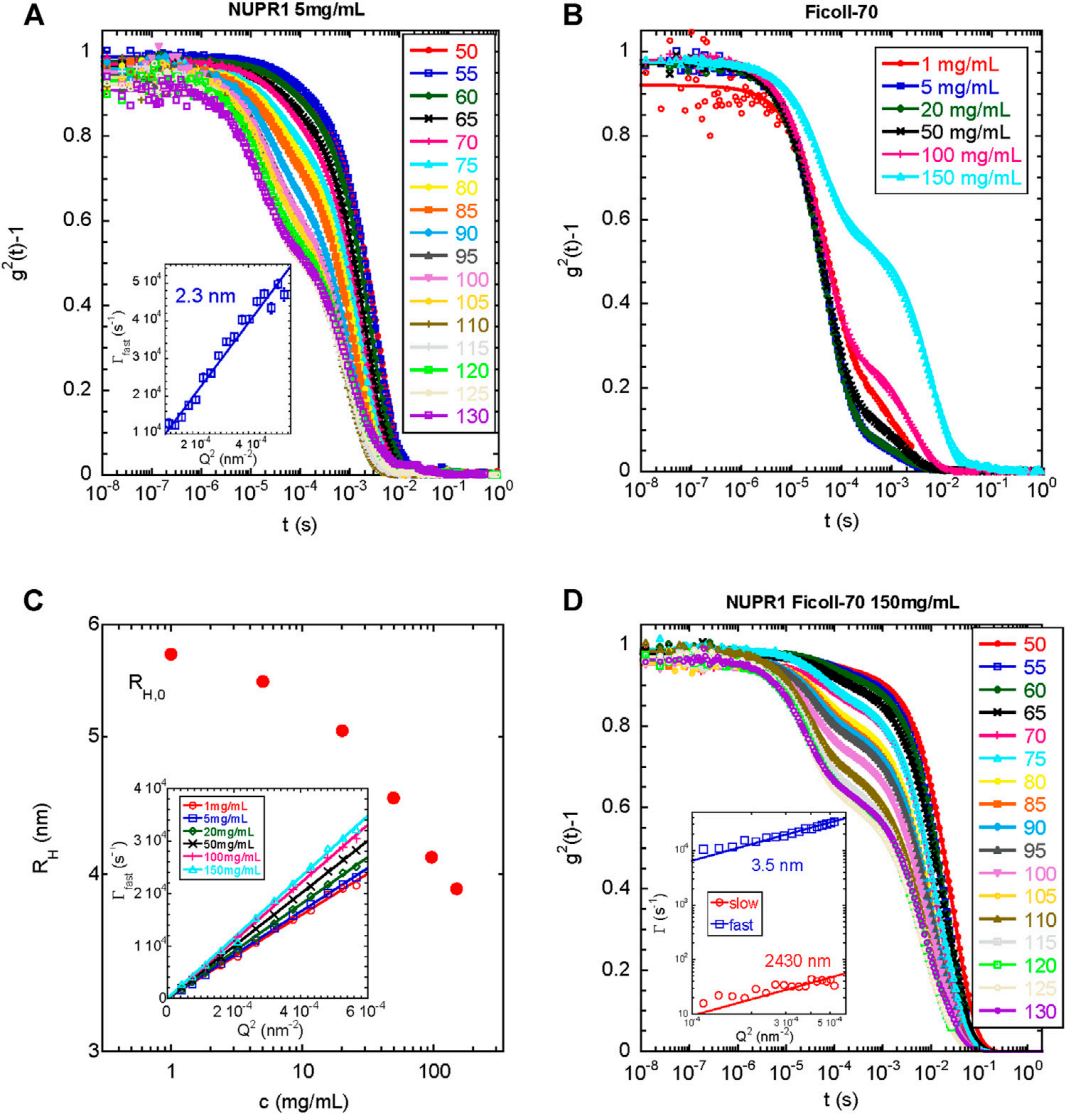
**DLS under different conditions for wild-type NUPR1: (A)** Intensity autocorrelation functions at different angles indicated for NUPR1 solutions at 5 mg/ml. Solid lines correspond to fits using Eq. 1 **(B)** Intensity autocorrelation functions at 90° for Ficoll-70 solutions in water from 1 to 150 mg/ml **(C)** Hydrodynamic radius as a function of the concentration of Ficoll-70. Inset: Fast relaxation rates obtained from fitting the correlation functions with Eq. 1. The solid lines represent the fits to 1/τ = *D*Q^2^ **(D)** Intensity autocorrelation functions at different angles of a mixed solution of NUPR1 4.3 mg/ml and Ficoll-70 150 mg/ml. Solid lines correspond to fits using Eq. 2. Inset: Q-dependence of the slow and fast relaxation rates.

Then, we explored the dynamics of the same protein solution by DLS. Figure 5 A shows the intensity correlation functions of the solution of isolated NUPR1, which clearly have two relaxation modes. The fast mode is due to the translational diffusion of the protein in dilute solution. Fitting of this data to Γ = [*k*_B_ *T*/6πη*R*_h_]Q^2^ yielded a hydrodynamic radius *R*_h_ = 2.3 nm (with an error of 5%), in good agreement with the SAXS result of *R*_g_ = 2.5 nm. The ratio of these two radii depends on the conformation of the polymer. For compact (spherical) globules, this ratio is 1.3, whereas for fully flexible random coils (see, for instance, Kok and Rudin, 1981; De la Cuesta et al., 2017) values of about 0.7–0.8 are found. In our case, *R*_h_/*R*_g_ = 0.92, which is much closer to the value expected for a disordered region. This supports the hypothesis of an unfolded conformation of the protein, though some degree of order cannot be ruled out.

We do not know the origin of the slow relaxation mode, which yielded an *R*_h_ = 261 nm (with an error of 5%); it did not disappear upon sample filtering and the resulting radius increased when the sample was left at room temperature for several days (for instance *R*_h_ was 359 nm (with an error of 5%), after 48 h at 25°C). We cannot rule out the presence of an impurity in the NUPR1 stock used, which tends to aggregate upon time, but it does not affect the measurements of the fastest relaxation mode, and it can be only detected by DLS. We can speculate that if the termini of NUPR1 had a tendency to form compact local conformations in the presence of crowders (as indicated by the EPR results, *EPR*), they might have an intrinsic tendency to form intermolecular interactions with the other polypeptide chains present in solution. Over the time, a partial entanglement of the polypeptide chain, which comes from possible transient catenane-like aggregates that do not longer completely dissociate (probably due to additional hydrophobic interactions), may show a slow diffusion. Such partial entanglement could be further speculated to be the natural precursor of a fluid-like aggregated spontaneously formed by NUPR1.

In the next step, we measured the light scattering dynamics in the Ficoll-70 solutions as a function of its concentration. We used only Ficoll-70 as a crowding agent, due to its distinct behavior with the A2C-MTSL species in the EPR experiments (Table 1). All solutions, in the range of Ficoll-70 concentrations from 1 to 150 mg/ml, exhibited two distinct relaxation modes (Figure 5B), with the second one disappearing after filtration with a PFTE filter. As we had already done with data for isolated NUPR1, we obtained the correlation functions by using Eq. 1. The fast relaxation rates showed a Q^2^-dependence (Figure 5C), and the diffusion coefficient increased with the polymer concentration, as it has been reported for semi-dilute polymer solutions (Zettl et al., 2009). The diffusion coefficient can be used to estimate the *R*_h_, which decreased as a function of the concentration (Figure 5C). At the most diluted concentration, the *R*_h_ of the synthetic polymer was 5.7 nm.

Finally, we explored the dynamics of a solution containing NUPR1 and Ficoll-70. To this end, we prepared a solution containing 4.3 mg/ml for NUPR1, and a concentration of 150 mg/ml of Ficoll-70. As it happened with the solutions of each isolated component, there were two relaxation modes in the correlation functions of the mixed solution. In this case, however, the sum of two exponentials did not fit properly the data, and instead, a suitable functional form was found to be a mono-exponential decay for the fast relaxation process together with a stretched exponential decay describing the slower relaxation mode (Eq. 2). Both the fast and the slow relaxation rates scaled as Q^2^, yielding hydrodynamic radii of *R*_h_ = 3.5 and 2,430 nm (with an error of 5%), respectively, (Figure 5D). The small radius was closer to that of isolated Ficoll-70, than to the radius of the protein (see above). The larger radius was too big to be ascribed to possible aggregates of the protein; however, it was larger, but of the same order of magnitude, than those aggregates observed for Ficoll-70 in 150 mg/ml solutions, which disappeared upon filtering, and thus, we ascribed that slow relaxation mode to aggregated species of the synthetic polymer. Therefore, we can conclude that the dynamics observed in the mixed system reflected primarily the dynamics of the crowding agent, which was slightly altered by the presence of NUPR1, and DLS was blind to the presence of the polypeptide chain. The fact that there was a modification of the dynamic behavior of the synthetic polymer (for the findings on the smaller radius) in the presence of NUPR1 suggests the occurrence of binding, thus confirming the other spectroscopic results.

## Discussion

The main conclusion from our work is that NUPR1 remained disordered in the presence of crowders, except for local effects at both termini, as shown by the joint use of EPR, NMR and CD, and therefore, its disordered nature is not substantially altered in a crowded environment. Only the N and C-terminal regions seem to be slightly affected by the presence of the crowders, as shown by EPR. Since different types of conformational behaviors have been revealed for IDPs in crowding conditions, these proteins have been recently divided in three different categories: (partially) foldable (able to assume a partial fold in crowded milieu), non-foldable (insensitive to crowding conditions) and un-foldable (IDPs which undergo unfolding due to crowded environment) (Fonin et al., 2018). Based on our findings, we speculate that NUPR1 belongs to non-foldable IDPs, since only partial local variations were observed with crowding agents at different concentrations.

Several studies have shown the usefulness to compare results from protein in aqueous solutions and those in a crowded environment, and it is clear that the *in vitro* mimicking of the complex crowder cellular interior is an oversimplification. Therefore the use of chemical compounds as mimickers of the complex cell environment is still a matter of debate. However, some IDPs, such as *α*-synuclein, prothymosin *α*, or the flagellar synthesis regulator in *Salmonella typhimirum*, FlgM, showed the same behaviour in the presence of crowders, as well as within a cellular environment (McNulty et al., 2006; Konig et al., 2015; Theillet et al., 2016; Bai et al., 2017; Fonin et al., 2018). Our control experiments in the presence of a more physiological-like environment than that provided by the crowders, as that happening in the presence of BSA, indicate that NUPR1 had a similar behaviour. Based on our results, we support the idea of using these synthetic polymers to reproduce *in vitro* the complex intracellular environment.

Theoretical simulations and different biophysical techniques such as NMR, SAXS, small-angle neutron scattering and single-molecule fluorescence indicate that IDPs, in the presence of high concentrations of synthetic polymers or under in-cell conditions (Qin and Zhou, 2013; Theillet et al., 2016), can have more compact conformations compared to those observed in isolation, although an expansion can also be observed under specific crowder concentrations, accompanied sometimes by an increase in helical content (Banks et al., 2018). On the other hand, the results obtained by using crowders with different IDPs have shown that, in all examples described so far ((Fonin et al., 2018) and references therein), the proteins remain disordered, not only in the presence of exogenous crowding agents, but also in a crowded cellular environment (Popovic et al., 2015).

The possible local compaction in a polypeptide chain, which results in reduced flexibility, has been always attributed to excluded volume effects, or alternatively to weak protein-crowder interactions, whereas the changes in solvent properties induced by the crowder have been usually disregarded (Breydo et al., 2015; Ferreira et al., 2016). However, it is known that the crowder may also alter water polarity, its polarizability and its acid/base properties, which in turn could also affect the conformational features of a protein (Kuztnezsova et al., 2015). In addition, due to the larger size of the crowders (when compared to the polypeptide chain), they can act as obstacles in the solution within the complex local-global dynamics and the translational movements of an IDP. Any possible intermolecular contacts could change very much the overall dynamics in the proximity of a high molecular weight polymer. Collisions of the polypeptide chain with the polymer network may change the dynamics of an IDP in a different manner compared to the way that the presence of the polymer affects solution viscosity. This is an additional aspect of crowding effects, not related to either excluded volume effects or the possible complex formation, associated with non-specific interactions between the polypeptide and the surrounding polymer (Jena and Bloomfield, 2005; Zhou et al., 2008).

In this study, we initially decided to investigate the local flexibility of NUPR1 at different concentrations of Dextran-40 and Ficoll-70. SDSL-EPR spectroscopy has been employed since it has the advantage to be insensitive to crowders at high concentrations, and it can probe minor dynamic states, which are very challenging to detect with other techniques. Our SDSL-EPR investigation of NUPR1 in buffer confirmed the intrinsic complex dynamic behavior of this protein, as the spectra revealed a line shape and spectral parameters typical of an IDP. EPR results on spin-labelled mutants demonstrated that some of NUPR1 ensemble population (up to ∼60%) possessed a less flexible dynamics in the presence of Ficoll-70 and Dextran-40 (or even in the presence of BSA (Supplementary Figure S10). The broadening of the EPR signals indicates the absence of an effective disorder-to-order transition of the system in crowded conditions. Our EPR data also suggest that the interactions between the protein and the two crowders induced distinct effects on the C-terminal and N-terminal regions of NUPR1. These results are in agreement with those described also for prothymosin *α*, TC1 and *α*-synuclein (Cino et al., 2012), which are also IDPs. The difference in local folding propensities at both termini, confirmed by our EPR experiments in the presence of TFE, supported the hypothesis that protein regions can be individually affected in different environments. Since the τ_c_ values derived from the EPR broader signals were still in the order of nanoseconds, and a fraction of MTSL-labelled protein still remained partially disordered with crowders, we can suggest that NUPR1 flexibility was mildly affected by the surrounding environment at both termini. We have previously observed that, upon binding to DNA or prothymosin α, changes could be observed in some of the local correlation times of NUPR1, which could not be ascribed to any polypeptide patch of the protein (Neira et al., 2019), while the rest of the protein remained disordered. It is tempting to suggest that, in the presence of crowder agents, we were also observing, as monitored by EPR, a change in the local correlation times of the polypeptide chain at both termini. Since we observed two NUPR1 populations with distinct correlation times in the EPR measurements, we do not believe that the changes affect the whole polypeptide chain. However, the dynamic properties of IDPs are difficult to interpret within the framework of a meaningful physical model; the different correlation times are determined by a sum of different contributions: polymer-like properties; non-uniform chain behaviors caused by the intrinsic (secondary) structural propensities; residue-dependent motions and long-range correlated movements (Parigi et al., 2014). Then, global and local motions are entangled and the great flexibility of NUPR1, at local and global frameworks, is a key-factor for its physiological activity. Although at this stage we cannot rule out any effect, it is tempting to suggest that the different behavior in the presence of the crowders could be related to the different charge distribution of NUPR1 (Supplementary Figure S1), with more positively charged residues at the C-terminal and more negatively charged at the N-terminal end. It is worth noting that local compaction at the N-terminal region has also been observed in *α*-synuclein in cellular conditions when it is modified at this end of the chain (Theillet et al., 2016). Although more examples with other IDPs should be described, we hypothesize that these proteins, within a cellular environment (or mimicker of such condition), start to acquire local compact conformation at their termini, without acquiring a fully folded conformation in the rest of their polypeptide chains.

In general, steric repulsion leads to compaction of IDPs, and the effect is larger for smaller crowders. This comes from the fact that, being equal the total volume fraction, larger crowders leave wider voids to be occupied by an IDP than smaller crowders. Therefore, we should have expected a larger correlation time for Dextran-40 than for Ficoll-70 for both labelled mutants. This happens for A2C-MTSL (Table 1), but it is not the case for N72C-MTSL mutant, for which we observed that in the presence of Dextran-40 the correlation time was lower than for Ficoll-70 (1.96 *vs.* 2.37 ns, Table 2). This suggests that the behavior of the MTSL probe, even in an entirely disordered protein such as NUPR1, differently affects the various regions along the whole polypeptide chain, and at position Asn72 there was an expansion in the presence of a smaller crowder. Therefore, we can conclude that subtle details in the NUPR1 sequence have a distinct influence also on the crowding effects and on the probe mobility (Miller et al., 2016). As a consequence, we cannot rule out that the charge distribution in NUPR1 (Supplementary Figure S1) could contribute to this different behavior observed at both its termini. In fact, it has been shown that the extent of crowding-induced compaction depends on the own features of the protein as well as on the concentration and size of the crowder (Miller et al., 2016).

## Conclusion

Disordered proteins are inherently complex systems with very specific features, and may sometimes have a rather unpredictable behavior in terms of accessible structural ensemble and conformational dynamics. This is especially true for NUPR1, a multifunctional IDP with an entirely disordered 82-amino-acid sequence and a high sensitivity to subtle local alterations in its conformation or changes in the surrounding environment. The results of this work show that crowding effects induce *in vitro* a locally compact, but still fully unfolded protein state. Findings obtained at two concentrations of Ficoll-70 and Dextran-40, and further comparison with the effect in the presence of different amounts of sucrose, show that this effect is crowder-dependent, and affects locally at both termini of the protein, although at a different extent. The effects of confinement and the presence of the polymer surface contribute to induce preferred local structural conformations that drive a conformational selection on the overall protein ensemble in solution. The outcome of this work is relevant to improve our understanding of the essential properties of NUPR1 and other IDPs under realistic physiological conditions, including their behavior in cellular environments.

## Data Availability

The original contributions presented in the study are included in the article/Supplementary Material, further inquiries can be directed to the corresponding authors.
