# Supplementary material for: Crowding Effects on the Structure and Dynamics of the Intrinsically Disordered Nuclear Chromatin Protein NUPR1
Source: Front Mol Biosci. 2021 Jul 5;8:684622. doi: 10.3389/fmolb.2021.684622 (PMC8287036; doi:10.3389/fmolb.2021.684622)
Supplement: Supplementary file 1 [file DataSheet1.docx]

Supplementary Figures

10 20 30 40 50 60

MATFPPATSA PQQPPGPEDE DSSLDESDLY SLAHSYLGGG GRKGRTKREA AANTNRPSPG

70 80

**GHERKLVTKL QNSERKKRGA RR**

**Supplementary Figure 1**. **Sequence of human NUPR1**

#

**Supplementary Figure 2**. **Far-UV CD spectra of isolated crowders: (A)** The far-UV CD spectra of Ficoll-70 at different concentrations. **(B)** The far-UV CD spectra of Dextran-40 at different concentrations. The experimental conditions are the same used in Figure 1 of the main text.





**Supplementary Figure 3**. **Far-UV CD spectra of labelled mutants:** The far-UV CD spectra of both MTSL-labelled mutants, and that of the wild-type protein.


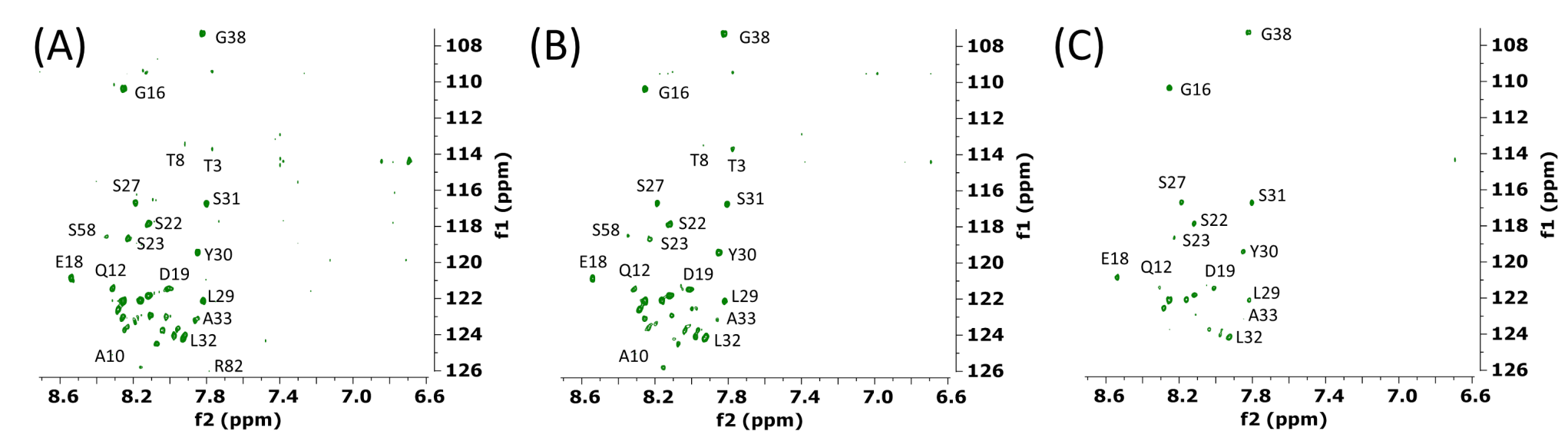


**Supplementary Figure 4**. **NMR spectra under different conditions for wild-type NUPR1: (A)** 2D ^15^N, ^1^H- TROSY-HSQC-NMR spectra of isolated wild-type NUPR1. **(B)** 2D ^15^N, ^1^H- TROSY-HSQC-NMR spectra of wild-type NUPR1 in the presence of 50 mg/mL Ficoll-70. **(C)** 2D ^15^N, ^1^H- TROSY-HSQC-NMR spectra of wild-type NUPR1 in the presence of 50 mg/mL Dextran-40. All spectra were drawn with the same lowest contour level. The cross-peaks which are not labelled in the center of the spectra correspond to overlapping of cross-peaks of two or more residues.

Ficoll-70

Dextran-40

**Supplementary Figure 5.** **Viscosity measurements**: Viscosity of Ficoll-70 and Dextran-40 aqueous solutions as a function of concentration. Experiments were carried out at in buffer Tris (50 mM, pH 7.0) at 25 ºC.


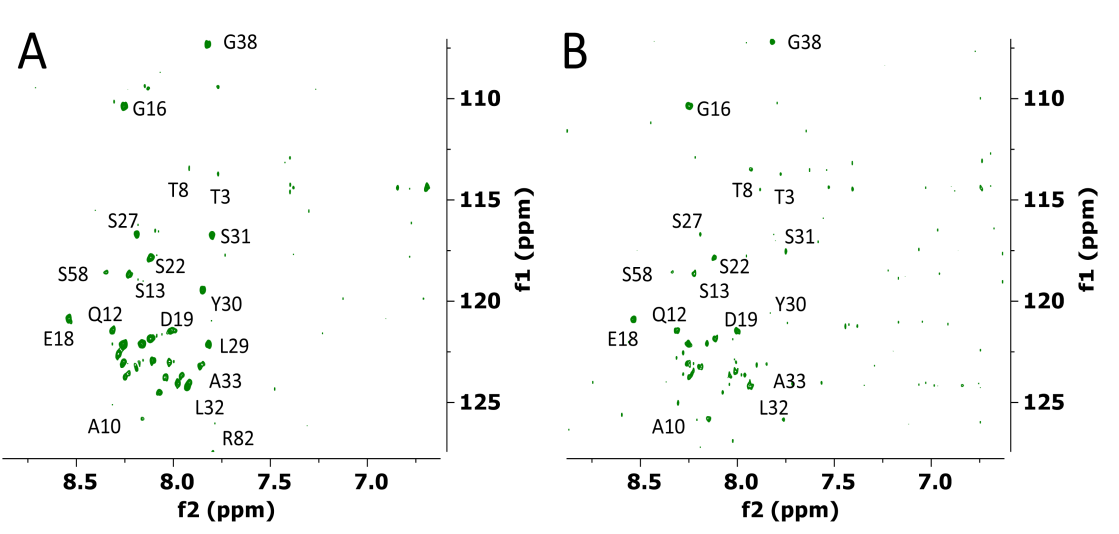


**Supplementary Figure 6**. **NMR spectra under different conditions for wild-type NUPR1: (A)** 2D ^15^N, ^1^H- TROSY-HSQC-NMR spectra of isolated wild-type NUPR1. **(B)** 2D ^15^N, ^1^H- TROSY-HSQC-NMR spectra of isolated wild-type NUPR1 in the presence of 50 mg/mL of BSA. The cross-peaks which are not labelled in the center of the spectra correspond to overlapping of cross-peaks of two or more residues.


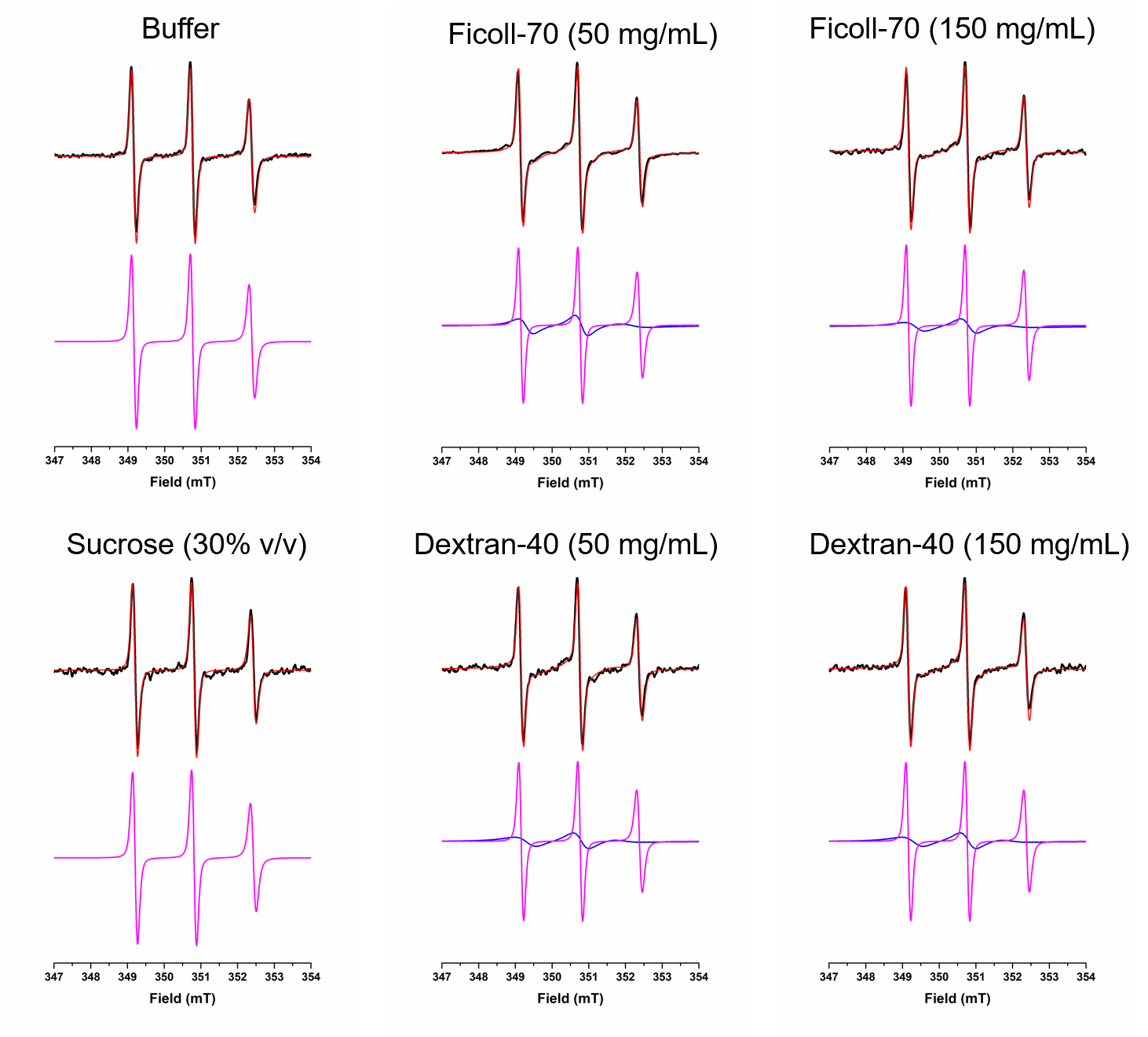


**Supplementary Figure 7.** **EPR spectra and simulations of A2C-MTSL**: Experimental (black) and simulated (red) X-band CW-EPR spectra of NUPR1 A2C-MTSL under different conditions. The components (sharp = pink and broad = blue) forming the total EPR signals are reported under each spectrum. Simulations were performed with SimLbel software (GUI of Easyspin).


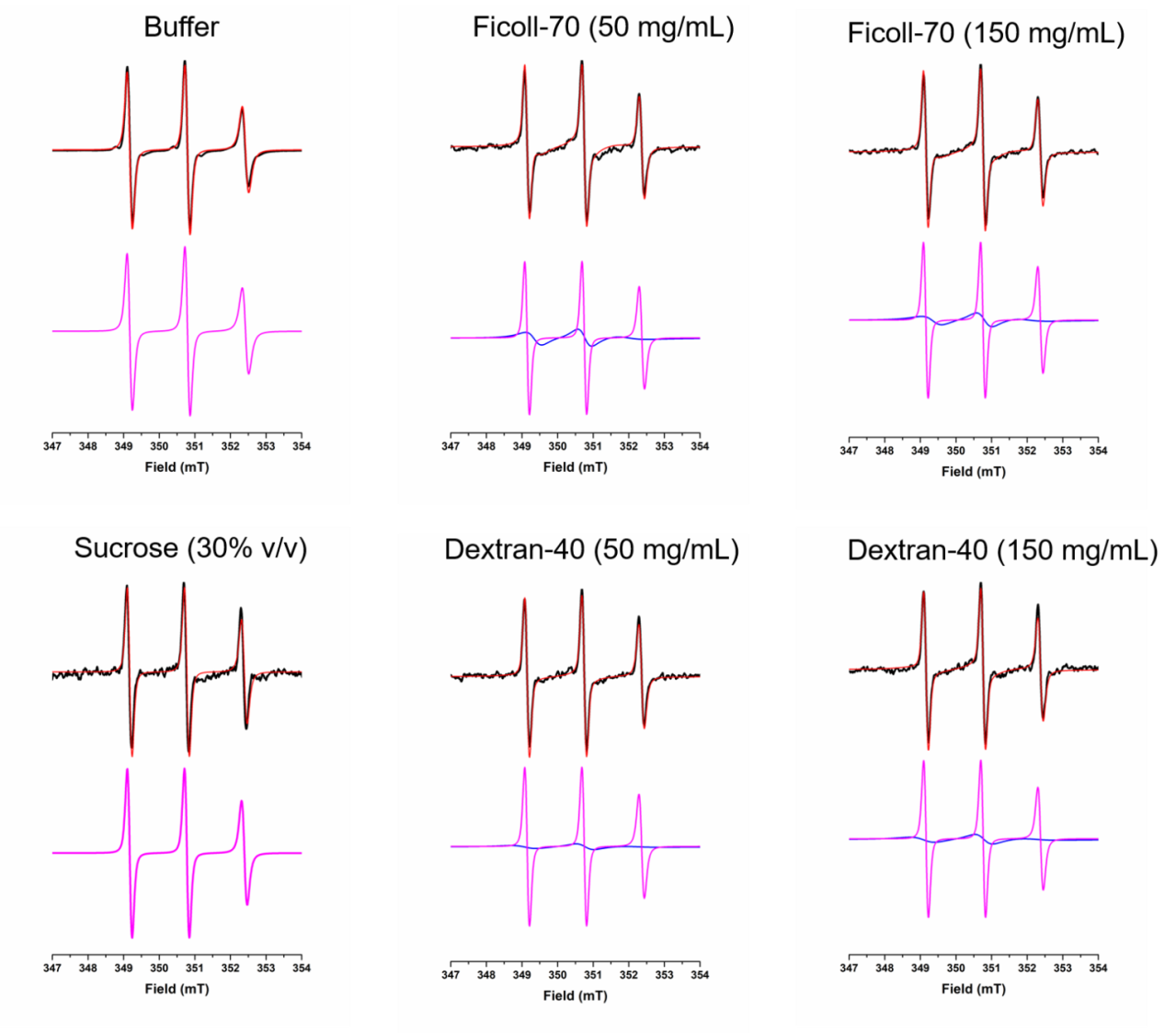


**Supplementary Figure 8.** **EPR spectra and simulations of N72C-MTSL**: Experimental (black) and simulated (red) X-band CW-EPR spectra of NUPR1 N72C-MTSL under different conditions. The components (sharp = pink and broad = blue) forming the total EPR signals are reported under each spectrum. Simulations have been performed with SimLbel software (GUI of Easyspin).


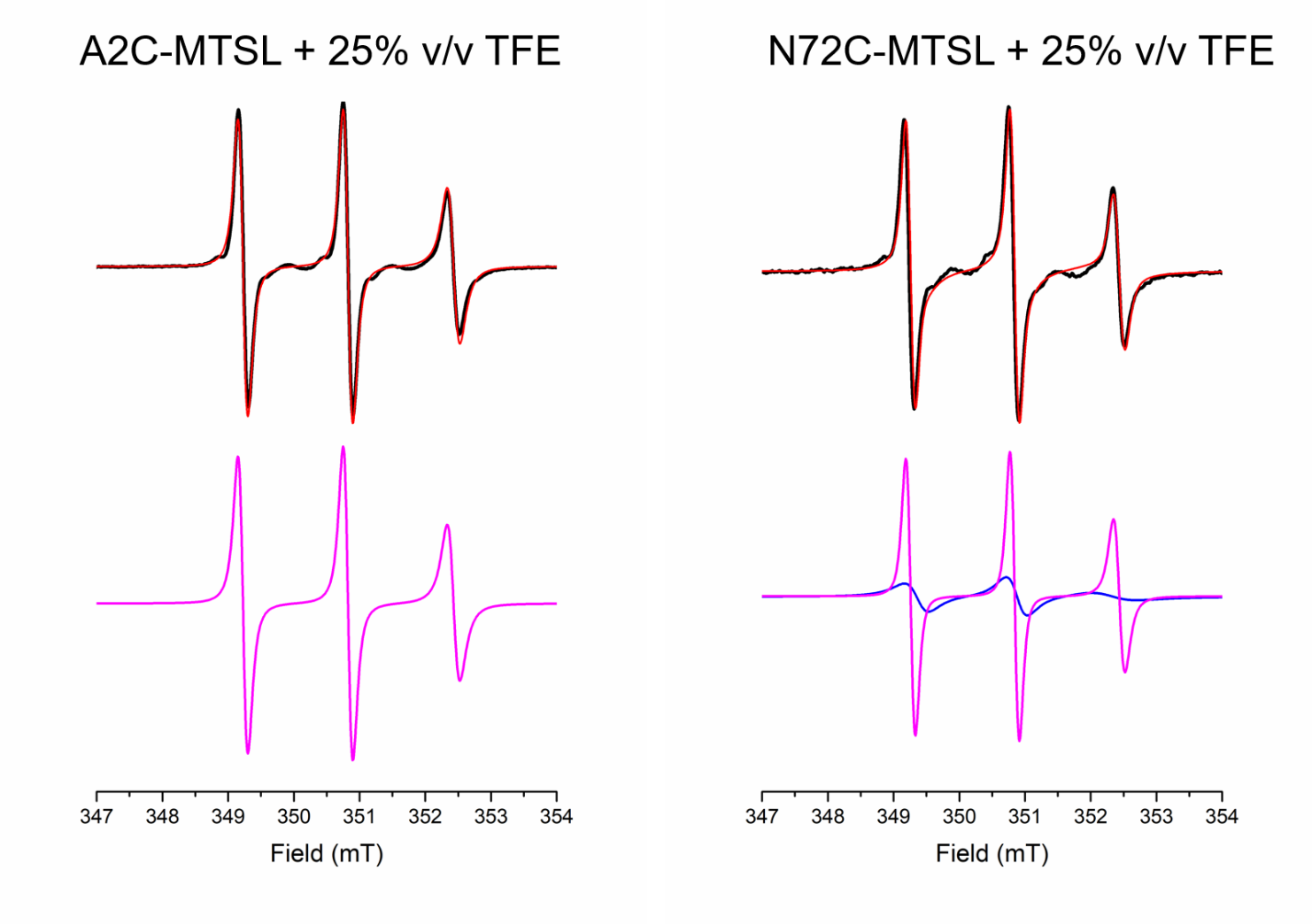


**Supplementary Figure 9. EPR spectra and simulations of MTSL-labelled mutants of NUPR1 in TFE**: Experimental (black) and simulated (red) X-band CW-EPR spectra at room temperature in 50 mM Tris (pH 7.2) with 25% v/v TFE. The components (sharp = pink and broad = blue) composing the total EPR signals are reported under each spectrum. Simulations have been performed with SimLbel software (GUI of Easyspin). Experiments were performed using a protein concentration of 30µM.


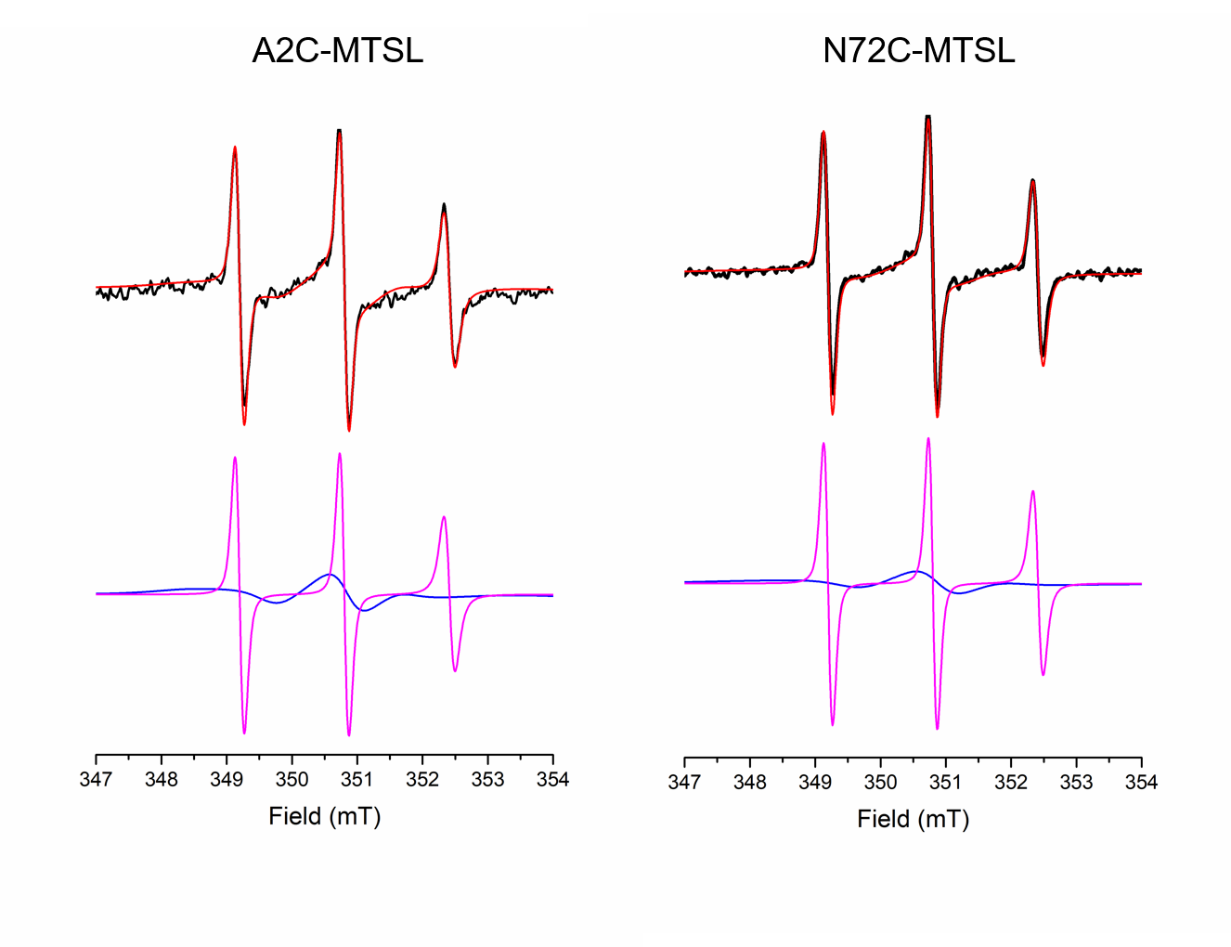


**Supplementary Figure 10. EPR spectra and simulations of MTSL-labelled mutants of NUPR1 in BSA**: Experimental (black) and simulated (red) X-band CW-EPR spectra at room temperature in 50 mM Tris (pH 7.2) with 150 mg/mL of BSA. The components (sharp = pink [31% with a τ_c_ = 0.16 ns and 43% with a τ_c_ = 0.12 ns respectively for A2C-MTSL and N72C-MTSL] and broad = blue [69% with a τ_c_ = 2.78 ns and 57% with a τ_c_ = 2.04 ns respectively for A2C-MTSL and N72C-MTSL]) composing the total EPR signals are reported under each spectrum. Simulations have been performed with SimLbel software (GUI of Easyspin). Experiments were performed using a protein concentration of 30µM. Data on the left are from A2C-MTSL and those to the right for N72C-MTSL.
